# 3D Team Care Management Trial: Study Design Features and Participant Characteristics

**DOI:** 10.1093/geroni/igaa057.2678

**Published:** 2020-12-16

**Authors:** Richard Fortinsky

**Affiliations:** University of Connecticut, Farmington, Connecticut, United States

## Abstract

This clinical trial was designed in partnership with a Medicare Advantage (MA) plan, with the goal of comparing two care management approaches for its MA policyholders age >65 living with cognitive vulnerability. To test the efficacy of the 3D Team, we are using a randomized design, with the comparison group receiving telephonic care management currently offered to MA members. In this presentation, detailed aspects of the study design, characteristics of the study population, and patterns of 3D team referrals will be explained. To date, 390 older adults and 306 informal caregivers are enrolled in the trial toward a recruitment goal of 576 older adults and 380 caregivers. Among older adults, 40% have depression only, 23% have dementia only, and the rest have more than one of these conditions and/or delirium. Most common referrals by 3D Team nurse practitioners are to the community health educator and to physical and occupational therapy.

